# Marginal adaptation of three-unit interim restorations fabricated by the CAD-CA systems and the direct method before and after thermocycling

**DOI:** 10.4317/jced.57920

**Published:** 2021-06-01

**Authors:** Leyla Sadighpour, Farideh Geramipanah, Mehran Falahchai, Hasan Tadbiri

**Affiliations:** 1 DDS, MSc, Associate Professor, Dental Research Center, Dentistry Research Institute, Department of prosthodontics, School of Dentistry, Tehran University of Medical Sciences, Tehran, Iran. Prosthodontic CCP Clinic, Department of Clinical Science, Faculty of Dentistry, University of Toronto, Toronto, Canada; 2 DDS, MSc, Professor, Dental Implant Research Center, Department of prosthodontics, School of Dentistry, Tehran University of Medical Sciences, Tehran, Iran; 3 DDS, MSc, Assistant Professor, Dental Sciences Research Center, Department of Prosthodontics, School of Dentistry, Guilan University of Medical Sciences, Rasht, Iran

## Abstract

**Background:**

Limited information is available regarding the marginal adaptation of three-unit interim restorations fabricated by different techniques from different materials. Also, the efficacy of computer-aided design/computer-aided manufacturing (CAD-CAM) systems for their fabrication is still questionable. This study aimed to assess the marginal adaptation of three-unit interim restorations fabricated by the CAD-CAM systems and the direct method from different materials before and after thermocycling.

**Material and Methods:**

A sound mandibular second premolar and a second molar were prepared for a three-unit all-ceramic restoration. Metal dies were fabricated to simulate a case of mandibular first molar missing, and were mounted in an acrylic block. Seventy-two three-unit interim restorations were fabricated by different techniques from different materials in six groups (n=12). In the first four groups, restorations were fabricated from Tempron, Visalys Temp, Unifast III, and Acropars by the direct technique, while the Amann Girrbach and Arum CAD-CAM systems and Ceramill Temp blocks were used in the last two groups. Marginal gap in each group was measured under a stereomicroscope at ×100 magnification. The specimens then underwent 5000 thermal cycles (5-55°C), and the marginal gap was measured again afterwards. ANCOVA and Bonferroni test (for pairwise comparisons) were applied for statistical analyses (α=0.05).

**Results:**

Amann Girrbach and Arum CAD-CAM systems were not significantly different regarding the marginal gap of restorations (*P*=0.999). Among the traditional interim materials, Acropars showed significantly higher marginal gap than others (*P*<0.001). No significant difference was noted between other traditional materials. CAD-CAM interim materials showed significantly smaller marginal gap than traditional materials (*P*<0.001).

**Conclusions:**

The CAD-CAM interim materials yielded superior marginal adaptation in three-unit interim restorations compared with traditional interim materials. The type of CAD-CAM system had no significant effect on the final marginal adaptation of restorations.

** Key words:**Dental marginal adaptation, interim dental prosthesis, CAD-CAM.

## Introduction

Fabrication of interim restorations is an important step for successful prosthetic treatments (1). Interim restorations are imperative to replace the lost tooth structure, biologically and mechanically protect the remaining tooth structure during the process of tooth preparation, and stabilize the position of the prepared teeth (2). Gingival health and pulpal protection of the prepared teeth are important points to consider in fabrication of interim restorations (3). Moreover, achieving accepTable esthetics, preventing the movement of the adjacent abutments, and maintaining a suiTable occlusion are among the other objectives behind the fabrication of interim restorations (4).

Interim restorations can be fabricated from polymethyl methacrylate (PMMA), polyethyl or butyl methacrylate, microfilled bisphenol A-glycidyl dimethacrylate, urethane dimethacrylate, and light-polymerizing resins (5). The primary monomer in the composition of these materials plays a role in their properties such as polymerization shrinkage, strength, and reaction temperature (6). Evidence shows that bis- acryl composite based temporary materials have higher color stability, lower polymerization shrinkage, and superior mechanical properties, compared with acrylic resins (7). Nonetheless, no material possesses all the required ideal properties, and the clinical selection should be based on factors such as cost, easy application, working time, esthetics, strength, and marginal accuracy. Although several materials are available for direct fabrication of interim restorations, the available materials used by the computer-aided design and computer-aided manufacturing (CAD-CAM) systems are limited, and are mainly composed of PMMA.

Interim restorations can be fabricated via different techniques. The direct (in-office) technique is suitable and cost-effective for the fabrication of a limited number of interim single crowns. Otherwise, this technique is highly time-consuming (7), and has shortcomings such as high polymerization shrinkage and thermal reaction (8). At present, aside from the conventional laboratory systems for indirect fabrication of interim restorations in a laboratory setting, the CAD-CAM systems can also be employed for this purpose. The advantages of the CAD-CAM technology for this purpose include automated fabrication process, higher quality of product, and shorter fabrication time (9). Also, the CAD-CAM systems minimize the possibility of errors in the fabrication process and risk of contamination (9). Considering the controlled milling process of the CAD-CAM blanks, the fabricated restorations have higher density and lower porosities. Moreover, the level of residual stress is minimized in them. All these factors contribute to the clinical success of restorations (10). It has been claimed that the technological advances in the new CAD-CAM systems and development of software programs have eliminated or minimized the existing problems in this respect. If such claims are true, the marginal adaptation of fabricated restorations should be excellent. However, the accuracy of restorations fabricated by the CAD-CAM systems depends on the quality and accuracy of the system, and therefore, can be variable in different systems (11). Some factors affecting the accuracy of CAD-CAM restorations include software limitations in restoration design, and hardware limitations of the camera, scanner, or the milling machine. Moreover, the experience of the clinician and technician can affect the final outcome of laboratory and chair-side CAD-CAM systems (12,13).

Ill-fitted interim restorations can mechanically irritate the surrounding tissue and aggravate bacterial plaque accumulation and subsequent periodontal problems (6,7). Obviously, any change in the gingival status can necessitate the replacement of final restoration or prolong the course of treatment. Therefore, evaluation of marginal adaptation is imperative to assess the clinical success of interim restorations (2). In 1966, Christensen reported that the acceptable marginal discrepancy was 2-51 µm for supragingival and 34-119 µm for subgingival margins (14). In 1971, Mclean and Von Fraunhofer reported that marginal discrepancy < 80 µm was hardly detectable in the clinical setting, and marginal discrepancy < 120 µm was clinically accepTable and successful (15). The accepTable amount of marginal gap for interim restorations is similar to that for permanent restorations, because parameters such as the health of the supporting tooth structures and dental pulp should also be considered (16).

The traditional materials and techniques used for the fabrication of interim restorations have been previously studied in terms of adaptation and fracture resistance (5,17-19). Also, several studies have compared the traditional methods with novel techniques such as CAD-CAM milling in terms of marginal adaptation (2,4,7,10,20-22). However, further information is required regarding interim restorations fabricated by the CAD-CAM technology and the effect of type of CAD-CAM system, in addition to the type of material, on their properties. Moreover, the results of available studies on this topic are controversial. On the other hand, to the best of the authors’ knowledge, the marginal adaptation of multi-unit interim restorations (a factor that can greatly impact on marginal accuracy) fabricated by different techniques has not been compared in any previous study. Therefore, the purpose of this study was to compare the marginal adaptation of three-unit interim restorations fabricated by two different CAD-CAM systems with different materials versus the direct technique before and after thermocycling. The null hypothesis was that no difference would be found in marginal adaptation of three-unit interim restorations fabricated by different techniques/materials, and thermocycling would not affect their marginal adaptation either.

## Material and Methods

In this *in vitro*, experimental study, 72 three-unit temporary restorations were fabricated by different techniques from different materials in six groups (n=12) as follows: In the first four groups, restorations were fabricated from Tempron, Visalys Temp, Unifast III, and Acropars by the direct technique, while the Amann Girrbach and Arum CAD-CAM systems and Ceramill Temp blocks were used in the last two groups (Table 1).

**Table 1:**
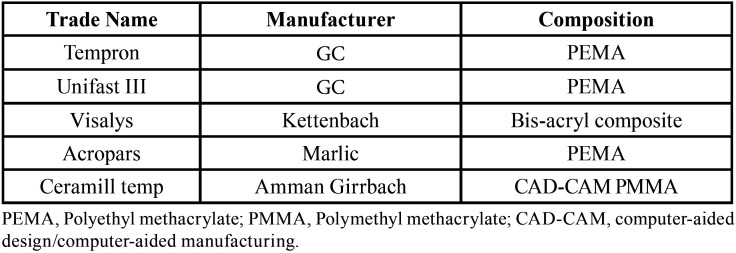
Information regarding interim materials used in this study.

**Figure 1:**
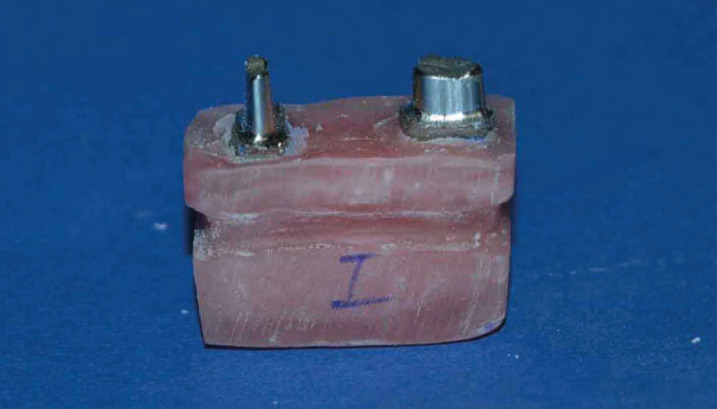
Fabricated model for simulation of mandibular first molar missing.

A mandibular second premolar and a mandibular second molar with no restoration or caries were first selected and underwent 2 mm of occlusal reduction and 1 mm of axial wall reduction with 4° taper and shoulder finish line by using a tapered flat-end diamond bur (ISO 856.014; Drendel + Zweiling Diamant GmbH, Lemgo, Germany) and high-speed handpiece under water coolant. The prepared teeth were mounted in a metal mold filled with wax measuring 3 x 1.5 cm. The teeth had 12 mm distance from each other (corresponding to the approximate mesiodistal width of a mandibular first molar) (9). The parallel position of the teeth was ensured by a surveyor. To prepare metal dies, the respective tooth was removed from the wax and an individual putty index (C-silicone Speedex; Coltene/Whaledent AG, Altstätten, Switzerland) was obtained from it. It was attempted to record the root area as well. The index was filled with acrylic resin (GC Pattern Resin; GC Corp., Tokyo, Japan), sprued, and invested with nickel-chromium alloy (Damcast NP; Damcast Dentalloy Corporation, Zhengzhou, China). Considering the number of specimens in each group (n=12 three-unit bridges), a total of 24 metal dies (12 second premolars and 12 second molars) were fabricated and used for the fabrication of specimens in the six groups. For standardized mounting of all specimens in acrylic resin, an index was obtained from the occlusal surface of the prepared teeth mounted in wax using zinc oxide eugenol and a wooden tongue blade. Metal dies were then mounted in auto-polymerizing acrylic resin (Technovis 4000; Heraeus Kulzer GmbH & Co, Wehrheim, Germany) to 1 mm below the cementoenamel junction using the index (Fig. 1). By doing so, the vertical and horizontal position of the abutments relative to each other, which could serve as a confounding factor, was standardized in different models. In order to prevent the polymerization shrinkage of acrylic resin during mounting, acrylic resin was applied incrementally.

For standardization of three-unit interim bridges, a wax model was fabricated on the prepared teeth as full-contour with 2 mm occlusal thickness, 1 mm axial thickness, and 4 × 4 mm connector dimensions, and cast with nickel-chromium alloy (Damcast NP). It was then completely seated on the tooth model. For better adaptation of the casting on the prepared teeth at the margins, the marginal area was precisely sealed with wax, and the excess material was removed. Next, an index was made from the final restoration using addition silicon with putty consistency (Panasil; Kettenbach GmbH and Co. KG, Eschenburg, Germany), which had stops at the peripheral margins. For the first four groups, a thin layer of petroleum jelly was applied on the dies with a microbrush, and then interim materials were mixed according to the manufacturers’ instructions, poured into the index, and the index was placed on the metal die. A 500 g weight was applied to fix the restoration on the die. During setting (prior to the exothermic reaction), the putty index was removed from the model and seated again. After setting, 10 min of time was allowed, and then excess material was removed by an acrylic bur under a loupe at ×3 magnification. Next, the specimens were polished using a prophy cup and pumice paste, and were then stored in water for 24 h. The putty index was fabricated separately for each material, and split in half by a scalpel in order to safely remove the interim restoration. After removal, interim restorations were inspected under a microscope (Axiolab; Carl Zeiss, Jena, Germany) at ×10 magnification for detection of voids or other defects. In case of presence of voids, the restoration would be replaced with a newly fabricated restoration. All procedures were performed by the same experienced operator.

For the fabrication of specimens by the indirect technique (CAD-CAM) in Amman Girrbach group, the mounted metal dies were scanned twice by a light scanner (Ceramill MaP-400; Amann girrbach AG, Koblach, Austria), once alone and once with the temporary restoration fabricated by the direct technique by using the Scan Spray (Scan dry; Dentaco GmbH & Co. KG, Essen, Germany). Next, the respective temporary restoration for each specimen was designed by superimposition of the obtained scans by the CAD design software (Ceramill Mind; Amann girrbach AG, Koblach, Austria). The same dimensions used in the direct technique were also used here (2 mm occlusal surface thickness, 1 mm axial thickness, and 4 × 4 mm connector dimensions). Cement space was considered 60 µm. The PMMA acrylic blocks (Ceramill TEMP light 71 L20nm; Amanngirrba AG, Koblach, Austria) were milled in a 5-axis milling machine (Ceramill; Amanngirrba AG, Koblach, Austria), and 12 specimens were prepared for this group as such.

In the Arum group, similar to the Amman Girrbach group, the scans were obtained by 3Shape scanner (3Shape D710; 3shape Copenhagen, Denmark). All restorations were designed with the same parameters as the restorations in the Amman Girrbach group according to the described method using the CAD software (3shape CAD software; 3shape Copenhagen, Denmark). Eventually, the PMMA acrylic blocks were milled by a four-axis milling machine (Arum 4X-100; Daewoon, Doowon ID, Daejeon, Korea).

**Figure 2:**
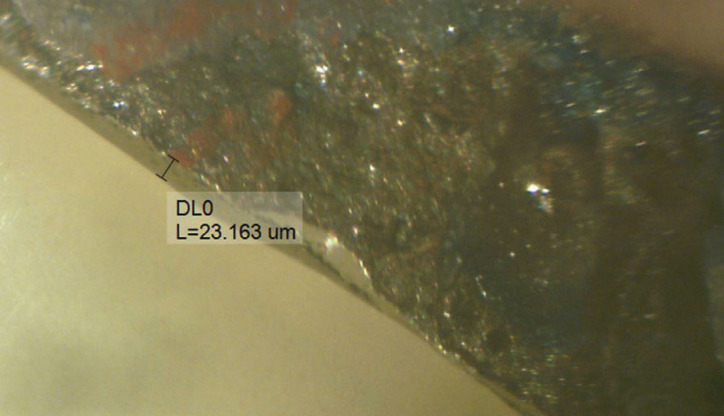
Measuring the marginal gap under a stereomicroscope at ×100 magnification.

Temporary restorations were placed on the respective dies and their marginal gap was measured under a stereomicroscope at ×100 magnification (Leitz optical stereomicroscope; Ernst Leitz GmbH, Wetzlar, Germany), which was equipped with a digital camera and a software program (Fig. 2). Since the direct-view technique was used, the abutment areas adjacent to the edentulous area (distal of second premolar and mesial of first molar) were disregarded since direct observation of gap was not possible in these areas. In order to standardize the sites of measurement of marginal gap, five points were marked at the midfacial, midlingual, mesiofacial, mesiolingual, and mid-mesial of the second premolar, and five points were marked at the midfacial, midlingual, distofacial, distolingual, and mid-distal of the second molar, 1 mm below the finish line by a permanent marker. The measurements were made at 15 points for each abutment (five main points plus points at the two sides of the midpoint of each surface with 100 µm distance). Next, the 15 obtained values were averaged, and the mean value was considered as the mean gap of each abutment. The mean gap of each three-unit restoration was calculated by averaging the mean gaps of the two abutments. All measurements were made by an experienced operator. Next, interim restorations underwent 5000 thermal cycles between 5-55°C with a dwell time of 20 s in each water bath. The marginal gap was then measured again. A device designed and used by Tjan *et al*. (23) was used to fix the restorations on the dies for measuring the gap.

Statistical analyses were carried out by using SPSS (SPSS Inc., IL, USA). ANCOVA was applied to assess the effects of type of material and type of tooth on marginal gap before and after thermocycling. The Bonferroni test was used for pairwise comparisons. Prior to this analysis, the homogeneity of variances was evaluated using the Levene’s test (Levene’s test=1.64, *P*=0.095). Since the assumptions were met, the fitness of the model was evaluated. The interaction effect of type of material and type of tooth was not significant and did not affect the adjusted R squared either. Thus, it was deleted from the model.

## Results

Table 2 presents the mean and standard deviation of marginal gap in the study groups before and after thermocycling. In ANOVA model, marginal gap before thermocycling was entered into the model as a confounder. By doing so, the effects of type of material and type of tooth on the marginal gap after thermocycling were adjusted. According to Table 3, assessment of the model fitness revealed significant effect of study groups on the marginal gap (*P*<0.001). However, type of tooth had no significant effect on marginal gap (*P*=0.343).

**Table 2:**
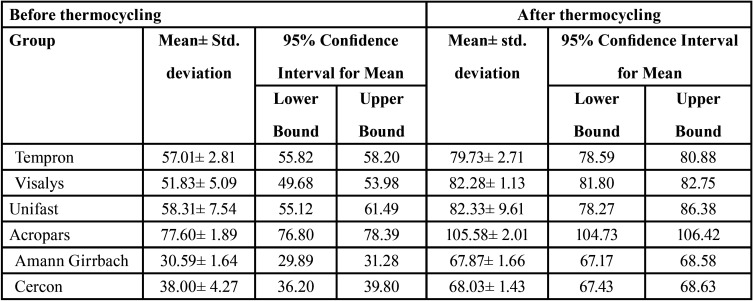
Marginal gap of three-unit interim restorations fabricated by different methods (n=12) before and after thermocycling (in micrometers).

**Table 3:**
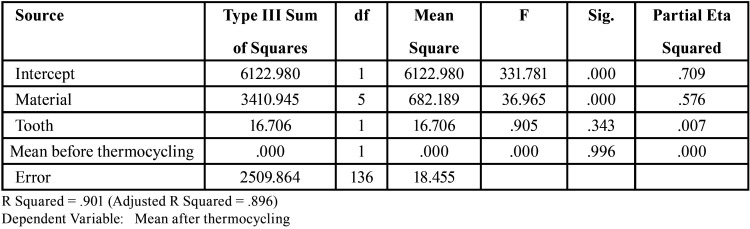
ANOVA model fitness results.

According to pairwise comparisons by the Bonferroni test, the marginal gap of restorations fabricated by the Amann Girrbach and Arum CAD-CAM systems was not significantly different (*P*=0.999). Comparison of the traditional and direct groups revealed that interim restorations fabricated from Acropars had significantly higher marginal gap than others (*P*<0.001). No significant difference was noted between other traditional groups (*P*>0.05). Moreover, the marginal adaptation of interim restorations fabricated by the CAD-CAM technology was significantly higher than that of traditionally fabricated interim restorations (*P*<0.001).

## Discussion

The current results indicated a significant difference in marginal adaptation of three-unit interim restorations fabricated by the CAD-CAM systems and the direct method from different materials before and after thermocycling. Thus, the null hypothesis of the study was rejected.

The marginal gap of restorations can be measured by several different techniques. The direct-view technique was employed for this purpose in this study. This technique measures the gap between the crown and die at the margin under a microscope at different magnifications. It does not measure the internal adaptation of restorations. This technique has drawbacks such as difficult differentiation between the tooth structure and the most inferior part of the finish line margin, and advantages such as being faster, and having lower cost since it does not require procedures such as sectioning of specimens. Also, this technique has lower risk of cumulative errors that occur in multi-step procedures (24,25).

The mean value of marginal gap in this study was 52-78 µm for the traditional materials and 31-38 µm for the CAD-CAM interim materials before thermocycling, and 80-106 µm for the traditional materials and around 68 µm for the CAD-CAM interim materials after thermocycling. The marginal gap values obtained in this study were lower than the values reported in previous studies (10,17,20,26), and were all within the clinically accepTable range (<120 µm). This difference can be due to the use of cement in the abovementioned studies, and also serial loading of specimens prior to thermocycling in some studies (17,26). According to some investigations, serial loading can increase the marginal gap (27). The obtained marginal gap values for the traditional materials in this study were almost similar to the values reported by Nejatidanesh *et al*. (5), for traditional interim materials. Nonetheless, it should be noted that direct comparison of the results of studies is not accurate because factors such as time of gap measurement, type of material used, tooth position, degree of taper of prepared tooth, type of adhesive, and technique of marginal gap measurement are widely variable in different studies (10). On the other hand, the abutment areas adjacent to the edentulous area were disregarded and the marginal gap in such areas was not measured in this study, which can affect the mean marginal gap of the crowns. Nonetheless, it did not affect the study objective, which was between-group comparison of marginal gap because the situation was the same for all groups. In this study, all interim restorations were fabricated by one operator. All measurements were also made by the same operator who was blinded to the restoration materials. This was done to minimize the laboratory errors.

The current results indicated that type of material significantly affected the marginal gap such that Acropars showed maximum marginal gap; however, no significant difference was noted between the other traditional materials. Nejatidanesh *et al*. (5) compared the marginal adaptation of restorations made of four interim auto-polymerizing resins by the direct technique. The maximum marginal gap was reported for Acropars while other materials showed values comparable to the values in our study. They concluded that higher marginal gap of Acropars can be due to its polymer to monomer ratio suggested by the manufacturer, which should be increased because polymerization shrinkage is influenced by a number of factors such as the filler volume and size, degree of conversion, type of monomer, resin flow, resin nature, and water sorption. Therefore, smaller size of monomer molecules and higher amount of monomer can lead to higher amounts of substrates for reaction in a certain volume, and subsequently increase the risk of shrinkage. Alternatively, it may be due to the exothermic polymerization reaction, which can lead to higher shrinkage during material cooling (5).

Literature is controversial regarding traditional materials. Young *et al*. (19) demonstrated that bis-acryl composite resin was superior to auto-polymerized PMMA due to lower polymerization shrinkage and absence of exothermic reaction. Conversely, Givens *et al*. (28) discussed that PEMA interim restorations had superior marginal adaptation compared with bis-acryl composite. However, another study as well as the current study showed that some traditional PEMA materials yielded comparable results to bis-acryl (5). Thus, it may be stated that in use of traditional interim materials, the manufacturer, adherence to the manufacturer’s instructions, and the technique used are more important than the type of material in achieving optimal marginal adaptation.

In this study, interim restorations fabricated by the CAD-CAM systems showed minimum marginal gap, which supported the previous results in this respect (2,4,9,10,13,21). The reason can be the absence of voids and milling of restorations out of pre-polymerized blocks since the polymerization shrinkage has already occurred in the processing of blocks (4). Another finding of this study was absence of a significant difference in marginal adaptation of restorations fabricated by the two CAD-CAM systems. Although the marginal gap of interim restorations has been previously compared, data regarding the effect of type of CAD-CAM system on marginal gap are limited. Memarian *et al*. (11) evaluated the marginal adaptation of three-unit zirconia bridges fabricated by Cercon, Amman Girrbach, and Zirkonzahn CAD-CAM systems. They reported that the type of CAD-CAM system had no significant effect on marginal adaptation of restorations. However, Hamza *et al*. (29) evaluated single-unit ceramic restorations and reported that the type of CAD-CAM system affected the vertical marginal discrepancy. Their results were in contrast to our findings.

The current results showed that type of tooth had no significant effect on marginal gap. To the best of the authors’ knowledge, the effect of type of tooth on marginal gap of interim restorations has not been previously evaluated, and adequate data in this respect are not available for the purpose of comparison. According to the results of the statistical model, thermocycling significantly affected the marginal gap such that in all groups (traditional and CAD-CAM), marginal gap significantly increased after thermocycling. Limited previous studies showed that marginal discrepancy increased after thermocycling, which can be attributed to the presence of voids, polymerization stress, residual and free monomers, and water sorption (10,26). Yao *et al*. (10) and Ehrenberg *et al*. (26) indicated that thermocycling significantly affected the marginal discrepancy of traditional interim materials such as PMMA and bis-acryl. However, in the study by Yao *et al*. (10) the single-unit CAD-CAM interim materials did not show a significant change in marginal discrepancy after thermocycling, unlike the traditional materials, which was in contrast to our findings on three-unit interim restorations.

This study was conducted on three-unit interim restorations. Limited data are available regarding such restorations. We tried to simulate the clinical setting as much as possible. The aging process was performed by 5000 thermal cycles, corresponding to approximately 4-5 years of clinical service (30). Nonetheless, this study had some limitations such as not using cement, not evaluating the internal adaptation of restorations, and difficult simulation of clinical conditions, which can affect the clinical interpretation of results. Thus, further studies with larger sample size and clinical trials are required to further elucidate this topic.
